# Diagnosis and management of acute appendicitis in 21 pediatric hematology and oncology patients at a tertiary care cancer center

**DOI:** 10.1038/s41598-021-90206-6

**Published:** 2021-06-09

**Authors:** Hannah von Mersi, Thomas Benkö, Heidrun Boztug, Michael Dworzak, Gernot Engstler, Waltraud Friesenbichler, Caroline Hutter, Karoly Lakatos, Georg Mann, Martin Metzelder, Roswitha Lüftinger, Herbert Pichler, Fiona Poyer, Leila Ronceray, Andishe Attarbaschi

**Affiliations:** 1 Department of Pediatric Hematology and Oncology, St. Anna Children’s Hospital, Medical University of Vienna, Kinderspitalgasse 6, 1090 Vienna, Austria; 2 Department of Pediatric Surgery, Medical University of Vienna, Vienna, Austria

**Keywords:** Cancer, Oncology

## Abstract

Acute appendicitis is a rare gastrointestinal complication of anti-cancer chemotherapy and hematopoietic stem cell transplantation. Among a cohort of 2341 hemato-oncologic patients at a pediatric tertiary care cancer center, we identified 21 patients (0.9%) with 23 episodes of acute appendicitis, based on pathological imaging of the appendix and clinical findings. Median age at diagnosis was 10.21 years. Types of underlying disease included acute leukemias (n = 15), solid tumors (n = 4), and aplastic anemia (n = 2). Clinical symptoms seen in > 1 case were recorded for all 23 episodes as follows: abdominal pain, n = 22; abdominal tenderness, n = 4; fever, n = 7; nausea, n = 2; emesis; n = 2; diarrhea, n = 5; and constipation, n = 2. Median leukocyte count at diagnosis was 0.5 × 10^9^/L, with a median of 0.1 × 10^9^/L for the absolute neutrophil count (ANC). All patients received broad-spectrum antibiotics and 18/23 (78%) patients underwent uneventful appendectomy after a median of 5 days and with a median ANC of 0.7 × 10^9^/L. Median duration until continuation of chemotherapy was 17 days for the 20 cases of appendicitis occurring during the patients’ disease course. Overall, 5/21 (19%) patients died including one related to the appendicitis itself which progressed to a typhlitis and was due to a fungal infection. The other fatalities were transplant- (n = 2) and leukemia-related (n = 2). Acute appendicitis is a rare and usually not life-threatening event in pediatric hemato-oncologic patients, which, if managed by prompt administration of broad-spectrum antibiotics (and antimycotics), can be safely followed by an elective (delayed) appendectomy, even before complete recovery of the neutrophils is achieved.

## Introduction

Acute appendicitis is a rare infectious gastrointestinal complication in children and adolescents undergoing anti-cancer polychemotherapy or allogeneic hematopoietic stem cell transplantation (HSCT)^1^. It may occur either alone or overlap with acute typhlitis, which is defined by the triad of neutropenia, abdominal pain and fever, as well as a thickness of the bowel wall of ≥ 0.3 cm^2–6^. Appendicitis is an inflammation of the appendix, which leads to an increased diameter of the organ to ≥ 0.6 cm and corresponding clinical signs. Given the rarity of acute appendicitis in the setting of immunosuppression, reliable data on the frequency, presentation, and outcome are scarce, with most data coming from case reports or small series^1,7–10^. Accordingly, risk factors and optimal management of acute appendicitis in hemato-oncologic patients have not yet been definitely established. In the present report, we retrospectively reviewed 21 pediatric hemato-oncologic patients with acute appendicitis diagnosed at a tertiary care cancer center (St. Anna Children’s Hospital) in Vienna, Austria, over a 16-years’ period.

## Patients and methods

We reviewed the medical records of all patients ≤ 18 years-old diagnosed with hemato-oncologic disorders and treated at our institution between 2003 and 2019 for the occurrence of an acute appendicitis. The diagnosis of acute appendicitis was based on the sonographically and, if available, on the via computed tomography (CT) or magnetic resonance imaging (MRI) confirmed thickness of the appendix of ≥ 0.6 cm. In addition, there had to be one or more clinical sign(s) suggestive of acute appendicitis such as abdominal pain, abdominal tenderness, fever, nausea, emesis, as well as diarrhea and/or constipation. Moreover, demographics and laboratory features, infectious agents, information on management, histopathology of the resected appendix, and outcome were analyzed. Data collection was approved by the ethics committees in Vienna (St. Anna Children’s Hospital and Medical University of Vienna), taking into account that all 21 patients were enrolled into national trials or registries of the underlying disorder, in which an episode of acute appendicitis had to be reported as a serious adverse event. Written informed consent for cancer and aplastic anemia treatment was obtained from all patients and/or their legal guardians and all methods pertinent to the present study were performed in accordance with the relevant guidelines and regulations.

### Ethical approval

Collection and assessment of the data were done with ethical approval.

## Results

The study population is shown in Table 1A–C. The male-to-female ratio was 13:8 and median age at diagnosis of acute appendicitis was 10.21 years (range 1.90–15.78 years). Types of underlying disease included acute leukemias (n = 15, 71%), solid tumors (n = 4, 19%), and aplastic anemia (n = 2; 10%). Four/21 (19%) patients had undergone a previous HSCT (median time since HSCT: 7.68 months, range 0.12–22.08 months), and 2/21 (10%) suffered from two episodes of acute appendicitis. Concerning the analysis of all further parameters, we always refer to the 23 episodes of acute appendicitis among the 21 patients. Clinical symptoms were: abdominal pain, n = 22; abdominal tenderness, n = 4; fever, n = 7; nausea, n = 2; emesis; n = 2; diarrhea, n = 5; and constipation, n = 2. None of the 23 episodes were life-threatening at the time of diagnosis. Acute appendicitis occurred in 15/23 (65%) and 7/23 (30%) cases while receiving systemic antibiotics and antimycotics, respectively, for other medical reasons.

**Table 1:** Initial characteristics and laboratory features (A, B) and therapy and outcome (C) of the 23 episodes in 21 patients with acute appendicitis.

Parameter	No. of pts. (n = 21)	Percentage
**(A)**		
Gender		
Male	13	62
Female	8	38
Age (years)		
Median	10.21	
Range	1.90–15.78	
< 10	9	43
≥ 10	12	57
Primary disease		
Acute leukemia^#^	15	71
Solid tumor^§^	4	19
Aplastic anemia	2	10
Previous allogeneic HSCT		
Yes	4	19
No	17	81
Time point after HSCT (months)		
Median	7.68	
Range	0.12–22.08	

Parameter	No. of episodes (n = 23)	Percentage
**(B)**		
Clinical symptoms at diagnosis		
Abdominal pain	22	96
Abdominal tenderness	4	17
Fever	7	30
Nausea	2	9
Emesis	2	9
Diarrhea	5	22
Constipation	2	9
WBC counts (× 10E9/L) at diagnosis		
Median	0.5	
Range	0.2–16.6	
ANC (× 10E9/L) at diagnosis		
Median	0.1	
Range	0–14.1	
< 0.5	17	74
< 1.5	20	87
Platelet count (× 10E9/L) at diagnosis		
Median	44.0	
Range	6.0–294.0	
Corticosteroids at diagnosis		
Yes	6	26
No	17	74
Imaging of appendix		
Sonography	23	100
Abdominal CT	3	13
Abdominal MRI	2	9
Thickness (cm) of appendix		
Measured	14	61
Not measured	9	39
Median	1.0	
Range	0.4–10.7	
Blood cultures		
Obtained	9	39
Not obtained	14	61
Positive	1 (MRSE)	
Negative	8	
Antibiotics at diagnosis		
Yes	15	65
No	8	35
Antibiotics given		
Piperacillin/Tazobactam	14	61
Amikacin	9	39
Teicoplanin	2	9
Vancomycin	5	22
Metronidazole	3	13
Ceftazidime	1	4
Netilmicin	1	4
Antimycotics at diagnosis		
Yes	7	30
No	16	70
Antimycotics given		
Voriconazole	4	17
Liposomal AmB	2	9
Fluconazole	1	4
Posaconazole	1	4

Parameter	No. of episodes (n = 23)	Percentage
**(C)**		
Appendectomy		
Yes	18	78
No	5	22
Histopathology		
Compatible with appendicitis	15	83
Infiltration with myeloid blasts	1	6
Not available	2	11
Antibiotic treatment		
Piperacillin/Tazobactam	17	74
Amikacin	17	74
Metronidazole	16	70
Vancomycin	10	43
Meropenem	9	39
Ceftazidime	9	39
Teicoplanin	6	26
Linezolid	5	22
Netilmicin	2	9
Others	8	35
Antifungal treatment		
None	7	30
Liposomal AmB	7	30
Fluconazole	7	30
Voriconazole	4	17
Posaconazole	2	9
Itraconazole	1	4
ANC (× 10E9/L) at the time of surgery		
Median	0.7	
Range	0–14.1	
< 0.5	7	39
< 1.5	11	48
Platelet count (× 10E9/L) at the time of surgery		
Median	113.5	
Range	11.0–297.0	
Transfusion of granulocytes (days)		
Yes	7	30
No	16	70
Median	5	
Range	3–12	
Therapy with G-CSF		
Yes	15	65
No	8	35
Time to appendectomy (days)		
Median	5	
Range	0–31	
Duration until restart of chemotherapy (days)		
Median	17	
Range	0–61	
Outcome		
Alive	18	78
Dead	5	22
HSCT-related	2	
Leukemia-related	2	
Appendicitis/typhlitis	1	
Follow-up of surviving patients (years)		
Median	6.75	
Range	0.16–12.71	

*HSCT* Hematopoietic stem cell transplantation, *No.* Number, *pts*. Patients, *WBC* White blood cells, *ANC* Absolute neutrophil count, *CT* Computed tomography, *MRI* Magnetic resonance imaging, *MRSE* Methicillin-resistant *Staphylococcus epidermidis*, *G-CSF* Granulocyte-colony stimulating factor.

^#^Acute lymphoblastic leukemia, n = 10; acute myeloid leukemia, n = 5.

^§^Osteosarcoma, n = 1; neuroblastoma, n = 2; Ewing’s sarcoma, n = 1.

All 23 cases had a sonography of the appendix, including three (13%) who had an additional CT and two (9%) cases with an additional MRI. For 14/23 (61%) cases, the sonographic measurement of the diameter of the appendix was available, with a median of 1.0 cm (range 0.4–10.7 cm). One patient whose appendix had a diameter of < 0.6 cm was included in our cohort because the acute appendicitis was confirmed by MRI. In the remaining 9/23 (39%) cases, morphologic alterations of the appendix, as described in the ultrasound reports, were clearly consistent with an acute appendicitis, but measurements of the diameter were lacking. Seven/23 (30%) cases had an ascites. In 5/23 (22%) cases, concomitant typhlitis (severe involvement of the terminal ileum, n = 2/5; moderate involvement of the surrounding area of the appendix, n = 3/5) was found, and in 2/23 (9%) cases, an inflammatory conglomerate tumor was described.

Median leukocyte count at diagnosis of the acute appendicitis was 0.5 × 10^9^/L (range 0.2–16.6 × 10^9^/L), with a median of 0.1 × 10^9^/L (range 0–14.1 × 10^9^/L) for the absolute neutrophil count. Seventeen/23 (74%) cases were in aplasia (< 0.5 × 10^9^/L) at diagnosis. With regard to the cases which underwent an appendectomy, median absolute neutrophil count was 0.7 × 10^9^/L (range 0–14.1 × 10^9^/L; aplasia, n = 7) and platelet count 113.5 × 10^9^/L (11.0–297.0 × 10^9^/L) on the day of surgery. Blood cultures were obtained for 9/23 (39%) cases, of which one was positive (methicillin-resistant staphylococcus epidermidis).

Therapy of appendicitis included antibiotics in all 23 episodes, with piperacillin/tazobactam (n = 17/23; 74%) and amikacin (n = 17/23; 74%) being the most frequently used and combined drugs. Meropenem (n = 9/23; 39%), ceftazidime (n = 9/23; 39%), metronidazole (n = 16/23; 70%), vancomycin/teicoplanin (n = 10/23; 43% and n = 6/23; 26%), and linezolid (n = 5/23; 22%) were also used, with metronidazole and the glycopeptide or linezolid usually administered additionally and the others replacing the initial beta-lactams. Sixteen/23 (69%) cases also received systemic antimycotics. Fifteen/23 (65%) cases received granulocyte-colony stimulating factor and 7/23 (30%) patients received granulocyte transfusions (median 5, range 3–12 transfusions).

Eighteen/23 (78%) cases underwent non-emergency appendectomy after a median of 5 days (range 0–31 days), with six patients having a laparoscopy and 12 patients having an open surgery. In one patient, a small bowel segment was additionally resected. Three of the seven patients with typhlitis or a conglomerate tumor did not undergo surgery at all, four had their operation after a median of 17.5 days. In 16/18 (89%) cases, histopathology of the resected appendix was available, describing a definite inflammation consistent with an appendicitis in 15 patients, and an infiltration with myeloid blasts in one case. There was no significant morbidity or fatality in any of the 18 operated patients. The remaining 5/23 (22%) cases were managed on a conservative basis at the discretion of the local treating medical team, which included antibiotics, antimycotics and supportive care. All but one patient survived the acute appendicitis. The patient who died had a progressive typhlitis most likely caused by a double fungal infection which was established during the multi-modality anti-infectious therapy of the patient (Aspergillus, not further specified, detected in a removed septic thrombus of the arteria iliaca communis dextra and Rhizopus microsporus detected by broad-spectrum polymerase-chain-reaction in peripheral blood).

The median duration until continuation of chemotherapy was 17 days (range 0–61 days) for the 20 cases of appendicitis, occurring during the patients’ disease course and no therapy modification was necessary for any of them. Overall, 5/21 (19%) patients died (fungal appendicitis/typhlitis, n = 1; HSCT-related, n = 2; ALL, n = 2).

## Discussion

This report on acute appendicitis as a complication of anti-cancer chemotherapy or HSCT represents one of the largest case series of this complication reported to date. We analyzed the data of 21 patients with 23 episodes which occurred in 2341 (0.9%) hemato-oncologic patients treated at a pediatric tertiary care cancer center over a 16-years’ period. We thereby confirmed that acute appendicitis is a rare event in hemato-oncologic pediatric patients.

The occurrence of appendicitis is assumed to be caused by the toxic effects of polychemotherapy on the gastrointestinal mucosa, neutropenia, and chemotherapy-associated constipation. Moreover, acute appendicitis might develop from obstruction of the lumen of the appendix, which can be triggered by coproliths, food residues, mucus, or scarring stenosis after an inflammatory process.

Our analysis shows that acute appendicitis is largely associated with the therapy of acute leukemias, school age, abdominal pain as the leading symptom, neutropenia at the time of diagnosis, and with an excellent outcome after an elective appendectomy, which can be performed safely and without significant morbidity in neutropenic patients after application of antibiotics for a few days. As acute appendicitis could also be rarely due to fungal infections, considering empirical anti-fungal therapy must be taken into account, especially, in cases doing poorly with broad anti-bacterial therapy only^11,12^. In addition, appendectomy performed in a timely manner can be diagnostically helpful with respect to this causative possibility.

Acute appendicitis can, albeit rarely, be life-threatening, in particular, if overlapping with typhlitis, as seen in our cohort with one fatality and reported in the literature^2–4^. Our study shows that abdominal pain is almost always found in patients with acute appendicitis, but the method of choice for diagnosis is imaging with ultrasonography, because abdominal pain may be absent during prolonged periods of neutropenia as seen in one of our patients and anecdotally reported by others^13^. Clinical differentiation of acute appendicitis and typhlitis is hardly possible and both may well occur together, as also observed in our cohort. Notably, fever as a symptom of the triad of abdominal pain, fever, and neutropenia, used for the definition of typhlitis, was only seen in 7/23 (30%) episodes of acute appendicitis^3,14^.

Although our analysis does not establish definite evidence, we demonstrated that an elective appendectomy can be performed safely in granulocytopenic pediatric patients^7,15^. This is in line with the study of Mortellaro et al., who suggest that surgical interventions within 24 h of diagnosis can be performed safely in neutropenic patients^9^. Nevertheless, the best timing of surgery remains a matter of debate. Although not possible in all cases, our policy was to operate as soon as possible in order to avoid a “locus minoris resistantiae”, which may cause a recurrent episode of appendicitis and/or typhlitis during the next phase of neutropenia as observed in 2 of our 21 patients. While the management of typhlitis comprises the early use of broad-spectrum antibiotics and bowel rest, appendectomy might be the treatment of choice for “pure” appendicitis^2,3,10,15,16^. Whether laparoscopic appendectomy is the preferred procedure in neutropenic children is discussed controversially. The limited number of reported cases preclude therapeutic recommendations, but it may have advantages over open surgery with regard to visualization of the entire abdominal cavity, wound complication rate, postoperative pain, and duration of postoperative stay^9^.

In conclusion, acute appendicitis is rare in pediatric hemato-oncologic patients, and, if managed by prompt administration of broad-spectrum antibiotics (and antimycotics), can be safely followed by an elective (delayed) appendectomy in most cases, even during aplasia.

## Abbreviations

CT: Computed tomography
HSCT: Hematopoietic stem cell transplantation
MRI: Magnetic resonance imaging
